# A Review of the Role of Re-Irradiation in Recurrent High-Grade Glioma (HGG)

**DOI:** 10.3390/cancers3044061

**Published:** 2011-10-28

**Authors:** Maurizio Amichetti, Dante Amelio

**Affiliations:** ATreP, Agenzia Provinciale per la Protonterapia, via Perini 181, 38122 Trento, Italy; E-Mail: amelio@atrep.it

**Keywords:** high-grade glioma, recurrence, radiotherapy

## Abstract

Despite the use of more effective multimodal treatments in high-grade glioma (HGG), the outcome of patients affected by this disease is still dismal and recurrence is a very common event. Many therapeutic approaches, alone or combined (surgery, drugs, targeted agents, immunotherapy, radiotherapy, supportive therapy), are available in the clinical armamentarium so far. The attitude of physicians is increasingly interventionist, but recurrent HGG still remains a very difficult scenario to be treated. Radiotherapy with different re-irradiation techniques is increasingly proposed as a therapeutic option with interesting results, even though the resulting duration of response is usually quite short. Most lesions re-recur locally, with inadequate identification and targeting of viable tumor being the most important cause of failure. Prognosis is affected by many patient-, tumor-, and treatment-associated prognostic factors. Radiotherapy is delivered with many advanced modalities: 3D-CRT, intensity-modulated radiation therapy, stereotactic fractionated radiotherapy, radiosurgery, and brachitherapy with or without chemotherapy administration. In order to evaluate the feasibility and efficacy of re-irradiation in this setting, we reviewed the PubMed and MEDLINE databases restricting the search to original reports published from January 1990 to June 2011. The search resulted in a total of 155 reports: 78 of them covering 2,688 patients treated with different irradiation modalities overall fulfilled the entry criteria. Radiation therapy demonstrated to be an acceptable option in recurrent HGG with good response rates and acceptable toxicity.

## 1. Introduction

High-grade glioma (HGG, WHO grade III-IV) is the most common primary central nervous system tumor in adults, accounting for more than 60% of all brain tumors [1]. Surgery and radiotherapy represent the cornerstones for their therapeutic management. Nevertheless, patients with HGG have a dismal prognosis and after initial treatment the majority relapse.

Tumor control and survival in patients with glioblastoma (GBM) have improved in the last ten years with the use of radiotherapy (RT) plus concomitant and adjuvant temozolomide (TMZ). In the recent EORTC/NCIC randomized trial, the reported median and 2-year survivals were 14.6 months and 27%, respectively; however, the majority of tumors recurred locally within a few months [2].

Most data suggest that in certain patients re-treatment will result in additional survival time and stabilization of neurologic deterioration [3,4]. Many approaches are currently available for the salvage treatment of patients with recurrent HGG after initial RT, including surgical re-resection, re-irradiation, or systemic agent(s) administration with chemotherapy probably being the most frequent treatment option, yet to date no standard of care exists.

A surgical approach can be employed in selected patients, but optimal resection is very difficult because of the extensive parenchymal infiltration of recurrent disease, and may be associated with a high risk of morbidity [5,6]. Reoperation may often be difficult due to the patient's medical condition and the potential for further neurological compromise [7]. Thus, only patients with well-accessible tumors and a good performance status are usually managed with this approach [8].

Chemotherapy has been the mainstay of treatment for patients with recurrent disease. However, available regimens are limited by the general poor conditions of these patients as well as the related development of side effects and mainly result in a modest palliation. Re-challenging with TMZ or switching to a non-conventional TMZ regimen has become a common practice [9]. More recently several targeted therapies such as anti-VEGF antibodies, EGFR, PKC/PI3K/AKT and integrin inhibitors have been introduced in clinical practice and in clinical trials with very preliminary results [10].

Re-irradiation is generally discussed controversially for the risk of toxicity. In fact, the high radiotherapy dose (about 60 Gy) usually applied to reduce the risk of in-field relapse, generally hampers the use of a second full-dose radiotherapy course. However, re-irradiation has been shown to be of value after local relapse. The literature provides consistent data supporting both the feasibility and the survival lengthening capability in comparison with supportive care only. Safe and effective re-irradiation of brain malignancies is challenging. Several approaches have been undertaken to improve the therapeutic ratio including external beam radiation therapy (RT), bi-dimensional (2D) or three-dimensional (3D) with/without combined hypoxic cell radiosensitizers, brachytherapy (BT), intensity-modulated radiation therapy (IMRT), fractionated stereotactic radiation therapy (FSRT) and stereotactic radiosurgery (SRS). Regardless of the treatment employed, the prognosis of this patient population is dismal; therefore, the treatment related toxicity and quality of life remains crucial when considering the therapeutic options. The aim of this review is to offer a survey on the efficacy of retreatment of HGG with different radiotherapy techniques.

## 2. Results and Discussion

### 2.1. Methods

Relevant articles regarding the irradiation re-treatment of high-grade gliomas were systematically searched in the PubMed and MEDLINE databases. The search was limited to articles in the English language and to those dealing with humans. Only studies published from the beginning of 1990 through the end of May 2011 and providing clinical results of ten or more patients were included. The selected keywords to accomplish the search included “high-grade glioma”, “glioblastoma”, “recurrent”, “radiotherapy”, “intensity-modulated radiation therapy”, “fractionated stereotactic radiotherapy”, “radiosurgery”, “brachytherapy”, “gliasite”, “boron neutron capture therapy”, “radioimmunotherapy”. The resulting papers were reviewed and prioritized by relevant content. Traditional reviews, editorials, case reports and letters of opinion were excluded. Data presented in abstract form were not included, even if they added valuable information. A secondary review was performed using the reference list of the selected articles in order to identify further relevant studies. In case of repeated publications from the same institution, only the most recent was used for the analysis. On the basis of the collected data, the possibility of undertaking a meta-analysis was evaluated.

### 2.2. Results

**Figure 1.**
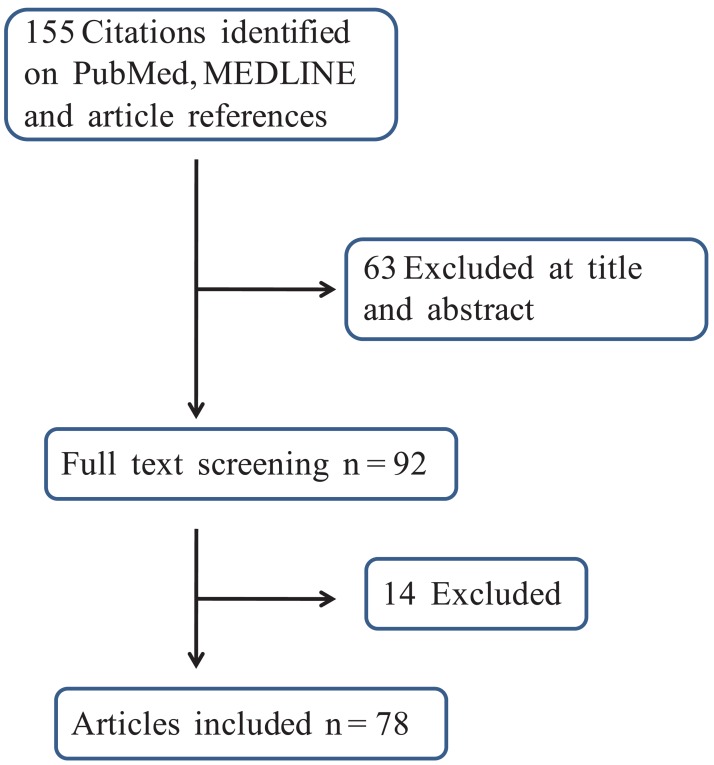
Study selection for inclusion into the review.

Overall, the search gathered 155 reports: 95 were retrieved from PubMed and MEDLINE while the article reference analysis provided a further 60 citations. According to the inclusion criteria, 78 updated papers were ultimately included in the analysis. The workflow used to generate the final number of studies is depicted in Figure 1

There were no prospective randomized trials and merely 17 (22%) prospective phase I-II studies. All the remaining papers were retrospective and only four had a control arm.

Considering that when randomized trials are not available and data mainly come from retrospective series pooling results is not recommended [93], the meta-analysis methodology was not applied. Hence, the following results are reported in the form of a narrative analysis.

Overall, the articles provided the clinical outcomes concerning 2,688 HGG patients. The techniques employed included conventional 2D/3D RT, FSRT, IMRT, SRS, BT, boron neutron capture therapy (BNCT), radio-immunotherapy (RIT), and pulsed reduced-dose-rate RT. The following sections provide an overview according to each technique.

#### 2.2.1. External Beam Radiation Therapy

There were only retrospective reports and non-randomized studies. Seven series were published between 1996 and 2009 with single series of 10–32 treated patients for a total of 144 patients (74 GBM and 70 HGG) reported (Table 1).

The patients had a median age ranging from 10 to 50 years and a median Karnofsky performance status (KPS) between 60 and 90. In two studies [13,14] a chemotherapeutic regimen with CCNU (lomustine) was combined and in another two about half of patients received some types of concurrent drugs [11,17]. Re-irradiation started after a median interval time of 14–38 months after the first irradiation. A cumulative biologically effective dose (BED) of 163.1–197.5 is calculated to be received by the re-irradiated patients. About half (seventy-one) of the re-irradiated patients had previously received some form of second surgery. The treatment was quite well tolerated: only a minor number of radionecrosis was reported [11,15-17], in two studies [11,17] some patients were re-operated, and in two studies [13,16] the use of corticosteroid was increased or maintained for a long period. The mean overall survival (OS) was 9.4 months. Better results were reported with the use of combined chemotherapy by Arcicasa *et al.* [14] and Hayat *et al.* [13], with a median OS after irradiation of 13.7 and 13 months respectively in comparison to 7–10.2 months of the other studies not using chemotherapy [11,12,15-17]. In general, neurologic toxicity was mild. The radionecrosis rate ranged between 4.5% and 30% (median, 6.5%). The reoperation rate was only reported in three studies [11,15,17], with a rate of 30%, 15.6% and 19.3% respectively.

#### 2.2.2. Fractionated Stereotactic Radiation Therapy

Twenty-four reports, published between 1993 and 2011, using FSRT as a method of re-irradiation, were retrieved; in 10/24 different types of chemotherapy were combined with radiotherapy (Table 2). A total of 773 patients were reported, with 575 cases of GBM and 198 of HGG. Median age was between 34 and 56 years and median KPS ranging between 60 and 90 (Table 2).

Dose of re-irradiation varied between a clear hypofractionated schedule with single doses ≥4 Gy [18-21,24,25,28-30,32,33,36,37], moderately hypofractionated schemes with the use of 3–3.5 Gy per fraction [22,23,35,39] or conventionally fractionated dose per fraction of 1.8 to 2.5 Gy [26,27,40-42]. Median total dose varied widely between 20 and 42 Gy, while median target volume, always defined by conventional morphologic imaging (CT/MR), was between 5.7 and 56.2 cc (median 24 cc). The mean OS for all the studies (radiotherapy and radiotherapy plus chemotherapy) was 9.9 months. Median OS was similar in patients treated with radiotherapy alone (range 6.7–16 months; median value 9.8 months) and with concomitant chemotherapy (range 7–14.5 months; median value 9.2 months). Overall, the concomitant administration of chemotherapy did not improve the results in comparison with radiotherapy alone. In five studies [21,24,28,30,33] some patients received salvage chemotherapy prior to re-irradiation. Only Vordemark *et al.* [28] reported no significant (p = 0.76) outcome difference between patients receiving re-irradiation up-front or after failed salvage chemotherapy; this issue was not evaluated in the remaining series.

Data regarding toxicity are available in 23 out of 24 studies. Thirteen series registered neither radionecrosis nor reoperation. Seven studies reported the occurrence of radionecrosis (range, 5–60%; median, 13.7%). Reoperation was registered in eight articles (range, 5.2–78%; median, 12%). Several prognostic factors present at the time of re-irradiation were individuated as statistically significant: age [39], PS [23,35], interval time to retreatment [29,39], dose of re-irradiation [22], tumor volume [21,27,32,39,42] and grade [23,28,31,32,34].

#### 2.2.3. Stereotactic Radiosurgery

Ultimately, 15 articles published between 1992 and 2011 met the inclusion criteria and were included in the review (see Table 3). All but five [44,47,49,53,55] were retrospective and recruited 594 HGG patients; 75% (n = 443) were GBM.

The median age ranged between 43 and 58 years, while median KPS varied between 70 and 90. The patients were re-irradiated after a median time of 4 to 19.8 months. The median target volume, always defined by conventional morphologic imaging (CT/MR), was between 2.7 and 30 cc, while the median re-irradiation dose ranged between 12 and 18 Gy.

Considering the treatment was always delivered in a single fraction, there was no concomitant chemotherapy administration. However, chemotherapy was employed as part of a re-treatment strategy in two reports [36,49]. Only two studies [49,52] reported that some patients received salvage chemotherapy prior to re-irradiation. The issue regarding the outcome difference between patients receiving re-irradiation up-front or after failed salvage chemotherapy was not addressed.

Overall, the use of SRS translated into median OS rates from re-irradiation of 5.3 to 12 months. Two studies pointed out values of 26 [53] and 30 [47] months, respectively. Only six papers provided data concerning progression-free survival (PFS) from re-treatment with an overall value ranging between 3.4 and 8.6 months. When reported, both OS and PFS were usually worse for cases harboring GBM compared to non-GBM HGG patients. Even though the treatment-related neurological side effects were generally mild, the reoperation rate varied between 3.5 and 23% with the radionecrosis rate up to 31.3%.

#### 2.2.4. Brachytherapy

The final selection provided 21 updated series employing BT as re-irradiation technique (see Table 4) published between 1991 and 2009. All but four [59,60,72,73] were retrospective, while merely two [68,71] had a control arm. The radiation sources included 125-I (13 studies), 192-Ir (4 studies) and 198-Au (1 study) with comparable results in terms of OS and PFS regardless the isotope used. The treatment was delivered with a high-dose or low-dose-rate. Depending on the article, implants were temporary or permanent. Three studies employed the so-called GliaSite system: it makes use of a silicone balloon catheter and an aqueous iodinated radiation source.

Overall, 877 HGGs were treated: median age of the patients ranged between 43 and 60 years, while median KPS varied between 70 and 90. In 11 series maximal safe resection mainly translating into gross/subtotal resection was attempted before re-irradiation. Only Patel *et al.* [70] pointed out a statistically significant better local control on multivariate analysis for patients who underwent a gross total resection. The median time before re-treatment ranged between 7.3 and 12.5 months. No studies demonstrated that such parameter could influence the prognosis. The median target volume, always defined by conventional morphologic imaging (CT/MR), was between 17 and 51 cc while the median re-irradiation dose ranged between 18 and 300 Gy. It is noteworthy that the latter total dose level was achieved by the means of a permanent low-activity 125-I source.

Chemotherapy was administered as part of a re-treatment strategy in two reports only [62,72]. Only in one study [60] some patients received salvage chemotherapy prior to re-irradiation. The issue regarding the outcome difference between patients receiving re-irradiation up-front or after failed salvage chemotherapy was not addressed. Overall, the use of brachytherapy translated into median OS from re-irradiation of 11.5 months (range, 5.5–18 months).

Only one study pointed out values above 24 months [69]. It is to note that such data deal with non-GBM HGG and that 92% of the patients received gross total resection before the implant. When reported, the PFS from re-irradiation ranged between 3.7 and 11 months, respectively. The comparison between GBM and non-GBM HGG patients was inconclusive due to the lack of consistency in the results.

In general, such results were achieved at the expense of mild neurologic toxicity. Nevertheless, the reoperation rate varied widely (9–56%) as well as the radionecrosis one (3–56%). Such results were comparable regardless the employed dose-rate as well as whether the type of implants was permanent or temporary.

#### 2.2.5. Other Techniques

The literature search gathered 11 other updated articles fitting the inclusion criteria: one concerning pulsed reduced-dose-rate RT [76], seven dealing with RIT [77-83], and three regarding BNCT [84-86]. Eight series were organized as a formal phase I or phase I-II clinical trial [77-83,86].

Overall, 290 HGG patients were treated: 54 received pulsed reduced-dose-rate RT, 180 RIT and 56 BNCT. Median age ranged between 45.5 and 58 years, while most series did not provide any data concerning either KPS or re-irradiation volume. When reported, the median time before re-treatment ranged between 5.9 and 18.2 months.

In the series dealing with pulsed reduced-dose-rate RT [76], the delivery of a median dose of 50 Gy translated into a median OS since re-treatment of 5.6 and 5.1 months for grade 3 and 4 tumors, respectively.

In the studies employing RIT [77-83], the delivery of an activity ranging between 10 and 120 mCi achieved a median OS from re-treatment of 6.3 [82] to 23.1 [81] months.

Finally, in the reports regarding BNCT the use of a dose varying between 13 and 73.9 GyE translated into a median OS from re-irradiation of 7 to 10.8 months.

In general, all these techniques proved to be feasible and safe being the treatment-related toxicity usually mild. However, two out of seven studies employing RIT registered paresis [82] and severe neurotoxicity [83] in 16.6% and 18% of patients, respectively.

## 3. Discussion

### 3.1. External-Beam Radiation Therapy (EBRT)

The potential of three-dimensional conformal radiation therapy (3D-CRT) for re-irradiation of selected intracranial tumors was studied in current practice at the beginning of the Nineties. Before, clinicians were hesitant to offer external beam re-irradiation with conventional fractionation at radical doses to patients who have previously been treated with full doses of radiotherapy (50–60 Gy) as part of their initial treatment. At that time, retrospective analyses [87,88] reported a risk of radionecrosis (RN) of the brain at the 5% level with doses ≥45 Gy with most cases occurring at 60 Gy, with a fraction-size dependent effect. Cases of delayed cerebral RN correlate most strongly with doses greater than 60 Gy delivered in 200-cGy fractions. These analyses were performed using whole brain or large partial brain portals only; therefore, extrapolation of this toxicity data to limited conformal partial brain irradiation was not warranted. The development of new 3D technology allowed the practical integration of CT and/or MRI into treatment planning and with this 3D planning process, conformal blocking techniques were applied more frequently to the re-irradiation of patients with recurrent gliomas. At the present time, the relationship between irradiation dose and volume is well established [89] and there is an agreement on the fact that more dose can be delivered to limited volumes. Moreover, the old concept of non-reirradiation has been overcome, and a second course of irradiation can be delivered keeping in mind the maintenance of a dose-memoire of 50% of the initial irradiation [90]. The literature data presented in the series using EBRT in the re-irradiation phase confirm the feasibility of a second treatment performed on limited fields. Quite satisfying rates of median overall survival of 6.1–13.7 months were obtained at a price of a low rate of RN or reoperation and neurological side effects (see Table 1).

### 3.2. Fractionated Stereotactic Radiation Therapy

Compared with conventional EBRT, stereotactic techniques (given by linacs or Gamma units), given as single fraction SRS or as FSRT, can deliver more localized irradiation with a steeper dose gradient between the tumor and the surrounding normal tissues reducing the risk of radiation induced complications. FSRT is advantageous in treating recurrent, previously irradiated, tumors, particularly when located in critical/eloquent areas. Its ability to divide the dose allows the therapeutic dose to be delivered over a number of fractions, while minimizing potential normal tissue toxicity. The divided dose should permit normal brain tissue to repair and give time for the tumor to re-oxygenate. The use of FSRT translated into mean OS and PFS of 9.9 and 6.4 months respectively. Analyzing the published literature on this subject (24 reports) we can observe a series of factors related to patient, tumor and to treatment potentially related to the results obtained. Some of them are discussed thereafter.

#### 3.2.1. Pre-Treatment Characteristics

##### 3.2.1.1. Interval Time

There is currently no consensus regarding the efficacy of salvage irradiation in patients who experience recurrence a short time after initial treatment. Grosu *et al.* [29] examining 44 patients with recurrent HGG found that an increased interval between initial diagnosis and recurrence was the most important prognostic factor associated with improved survival after re-irradiation. In contrast to this, Mayer and Sminia [91] reviewing ten years of re-irradiation studies did not find a correlation between time interval and prognosis. The study of Fogh *et al.* [39] did not demonstrate an inferior survival rate in patients who experienced a relapse within six months of primary treatment. The large number of patients examined in this study could have allowed to more accurately assessing this issue. This finding is of critical importance, considering that the eligibility in clinical trials is usually limited to patients who have survived at least six months from initial treatment.

##### 3.2.1.2. Patients' Performance Status

Karnofsky performance status (KPS) ≥70 at the time of recurrence was the strongest predictor for survival in some reports [23,35,92]. It is to note that KPS, (with tumor grade and age) is also a common independent variable predicting the longevity of patients with high-grade gliomas at initial diagnosis.

##### 3.2.1.3. Tumor Volume

Mixed findings regarding the influence of tumor volume on survival have been reported and are probably due to the different radiation doses, schedules and multimodal therapy used. Lederman *et al.* [21] showed that patients with tumor volumes <30 mL survived longer. In another study, tumor volumes <20 mL were associated with better response [22]. In other reports, tumor volume did not influence survival of patients after FSRT [23,35,92]. The volume is reported in different ways: as the tumor volume (GTV) in cm of diameter or in cc or as the irradiated volume (PTV). For this reason the values are not easily comparable. Target volume can also determine the risk of side effects. In the study of Ernst-Stecker *et al.* [32], some patients experienced an increase of edema with the need for an increase of steroid medication during the follow-up while GTV did not increase at imaging. The re-irradiated volumes in these cases were clearly higher than the median volumes treated.

##### 3.2.1.4. Tumor Grade

Grading is a well-known prognostic factor in the treatment of high-grade gliomas at diagnosis. The role of the grade of the primary tumor or at recurrence in re-irradiated patients is, instead, more controversial. WHO grading, both that determined at initial diagnosis of glioma and the most recent before SFRT, showed a significant impact on survival in the study of Vordemark *et al.* [28], while in other studies done by Shepherd *et al.* [20] and Cho *et al.* [23], only initial grade or grade at recurrence had a statistical significant value, respectively.

#### 3.2.2. Treatment Characteristics

##### 3.2.2.1. Dose of Radiation

In the majority of studies published in the literature investigating the effectiveness of FSRT, a relatively large dose per fraction (usually ≥5 Gy/fraction) is used in the hypofractionated regimens. Data showed that higher total doses of FSRT result in an improved survival [19,23,26,31]; but at the same time, doses greater than 40 Gy have been associated with increased toxicity with higher rates of re-operation [18,20], indicating a small therapeutic window. Similar data are reported with the use of high-dose single fractions of 5- to 6-Gy [21,25] with increased long-term toxicity in late-responding tissue in other disease sites. Fogh *et al.* [39] used 3.5 Gy fractions to 35 Gy and reported no grade 3 toxicities or re-operation secondary to toxicity, providing additional support that this moderate level of dose and fraction size is well tolerated [22,23].

FSRT given in small fractions of 2–3 Gy enables the precision and accuracy of SRS, while maintaining the radiobiological advantages of fractionation in terms of tumor control and protection of surrounding normal brain tissue [23,41].

##### 3.2.2.2. Combination with Chemotherapy

Although the role of chemotherapy combined with irradiation has been well established for patients with newly diagnosed primary GBM [1,2], there is a paucity of data reporting on the combination of chemotherapy and conventional [13,14] or SFRT [19,21,29, 30,33,34,37,40,41] for recurrent gliomas.

In the study of Fogh *et al.* [39], SFRT was associated with favourable survival benefit independent of second surgery or concomitant chemotherapy. Although it was not a randomized trial, the study did not demonstrate a survival advantage in combining chemotherapy with FSRT at recurrence compared with patients who received FSRT alone.

SFRT plus concomitant TMZ for patients with recurrent GBM is a feasible treatment associated with low toxicity; however, the survival benefits are modest [41]. In the study of Minniti *et al.* [41] patients with methylated MGMT and longer stable disease after primary standard chemoradiation have the better outcome, suggesting that methylation status of MGMT promoter also retains its prognostic value in recurrent GBM.

In general, the data of our review show similar median overall survival, comparing studies using FSRT alone or in combination with different schemes of chemotherapy, ranging between seven and fourteen months, considering also the different grades (III and IV) reported together in some series. The potential advantages of combined chemoradiation schedules to further improve outcome in patients with recurrent GBM and HGG need to be further explored.

##### 3.2.2.3. Toxicity

Because of the high total dose applied during the initial irradiation (usually near 60 Gy), the role of re-irradiation is largely debated regarding the risk of acute or late toxicity.

Radionecrosis, based on clinical features that included deterioration of neurologic deficits (without tumor progression) associated with abnormal findings on imaging such as positron emission tomography (PET), magnetic resonance spectroscopy (MRS), and/or other imaging studies (MRI or CT), is the most important side effect in this sense.

Many studies reported the development of RN after re-irradiation [19-21,25,31,41]. In several series patients were also reoperated after FSRT [20,21,23,25,31,37]. Patients who underwent surgical resection after FSRT often demonstrated radiographic progression confirmed by pathology, indicating these patients underwent re-operation mainly because of tumor progression rather than treatment-related effects.

### 3.3. Stereotactic Radiosurgery

Stereotactic radiosurgery is a non-invasive irradiation modality that can be delivered with gamma knife (Elekta, Stockholm, Sweden), Cyberknife (Accuracy, Sunnyvale, CA, USA), or specially adapted linear accelerators without relevant dosimetrical differences. It is a highly conformal, precise and accurate technique. Thanks to its features, stereotactic radiosurgery allows delivery of steep dose gradients, which translates into the sparing of the surrounding tissues. However, considering that treatment-related toxicity increases with target size as well as increased delivered dose, the lesions amenable by SRS are usually small.

These features best fit with small and round shaped relapsed HGGs, and automatically limit its wide use in this clinical scenario. Therefore, the large number of series we retrieved on its use is not surprising. However, due to the above-mentioned features, it is noteworthy the strict application clinical setting. In general, suitable patients were fairly young, with a high KPS and small relapses while the prescribed dose had very limited variations. From this standpoint, it is not surprising that all the series provided very consistent outcomes. Accordingly, such homogeneity hampered the detection of well-defined prognostic factors. Concerning OS, only few series pointed out the prognostic value of WHO grade [23,46,53], younger age [23,45,46] as well as KPS [23,46,47] while only four articles [23,45,47,53] revealed the role played by the treatment volume. However, with this respect, a clear volume-cutoff cannot be defined from literature data. Interestingly, the timing of re-irradiation does not seem to be of prognostic value. Based on the reported data, the risk of radionecrosis (up to 31%) should not be underestimated, hence the patient should be carefully selected.

### 3.4. Brachitherapy

Interstitial brachytherapy employing radioactive sources has been performed in recurrent HGGs because its higher spatial dose localization can improve the therapeutic ratio. Several sources such as 125-I, 192-Ir and 198-Au were employed to deliver high-dose or low-dose-rate irradiation as well as permanent or temporary implants ultimately generating great intra-modality variability. Regardless of the dose-rate and type of implant, the placement of multiple sources in the proximity of a resection cavity or relapsed tumor is challenging, and optimal dose distribution may be consequently difficult to be achieved [94]. However, the use of low-dose-rate interstitial BT could reduce the rate of severe complications in comparison with high-dose-rate implants. From this standpoint, a novel alternative temporary BT system (Gliasite, Cytic Surgical Products, Palo Alto, CA, USA) can overcome the limiting factors of conventional interstitial BT. In fact, working as a single spherical source of low-dose-rate radiation it can homogenously deliver a steep dose gradient around the surgical cavity.

This relevant variability, on the one hand significantly increased the volume of experience on BT re-irradiation of HGG; on the other hand, it decreased the possibility to address all the issues dealing with this technique. As a consequence, several topics such as the optimum prescribed dose, dose-rate, isotope, and implant modality have yet to be properly clarified.

In general, BT treatment of relapsed HGG can improve the survival with the high cost of radionecrosis. The capability of larger target irradiation with respect to SRS can only in part justify these data, while the promising results have to be interpreted in the light of the relevant reoperation rate before BT.

Moreover, it must be noted that, similarly to SRS, patients offered BT represent a highly selected population due to their favourable features (age, performance status, target volume). Considering the consequent patient homogeneity, the reviewed studies provided consistent results that ultimately hampered the detection of well-defined prognostic factors. With this regard, age [45,63,69,70,72], KPS [59,60,63,64,66,71,72,74,75] and treatment volume [64,69,71] demonstrated again the strongest predictive value.

### 3.5. Other Techniques

The literature search also provided data concerning three further techniques: pulsed reduced-dose-rate radiotherapy, radioimmunotherapy, and boron neutron capture therapy.

Regarding the first modality, the reduction in the delivery dose-rate might exploit differences between normal and malignant cells, allowing normal tissues to repair sublethal damage [76]. Moreover, splitting each fraction into a number of subfractions takes advantage of a second radiation phenomenon known as low-dose hyper-radiosensitivity [76].

Conversely, RIT achieves elevated local drugs concentration for a protracted time by locally delivering the chemotherapy compounds. Moreover, tissue-specific monoclonal antibodies labelled with high-energy β-emitting radionuclides can destroy a large number of tumor cells [80].

BNCT is based on the nuclear capture reaction that occurs when nonradioactive boron is irradiated with neutrons of sufficient thermal energy to yield high-energy α particles and lithium nuclei. The effect of α and lithium is limited primarily to boron-containing cells. The modality success is dependant upon a selective uptake of sufficient amounts of boron into cancer cells compared with normal tissues. Preferential uptake of boron into cancerous tissue is achieved using boron carriers [86]. Apart from these theoretical considerations only few data (overall, 290 patients) exist on the use of such techniques in recurrent HGG. Each modality proved to be safe and feasible while clinical outcomes are consistent with the series employing “conventional” re-irradiation modalities. However, considering that enrolled patients often received such techniques at their second or third relapse, they deserve further investigation as first-line re-treatment.

### 3.6. Considerations on Different Re-Irraddiation Techniques

Conventional fractionated radiotherapy using 3D-CRT is an outpatient-based, non-invasive approach that takes advantage of the properties of a standard fractionation schedule as well as of non-complex technique. However, it cannot best decrease the dose to neighbouring tissue. Therefore, the use of 3D-CRT, either alone or combined with chemotherapy, allows the delivery of relatively low dose in this clinical scenario and is not able to shorten the number of weeks of treatment. The few published data concerning patient re-irradiated by this technique pointed out acceptable side effect rates, whereas the clinical outcomes were not meaningful. To date, this technique should be employed to deliver short-course palliative re-irradiation in patients with worse prognostic factors.

With the advent of relocatable frames, it is possible to exploit the radiobiological advantages of fractionation with the possibility of improving the therapeutic ratio. FSRT can be delivered as an outpatient-based, non-invasive approach that takes advantage of the stereotactic precision. Hence, large tumors, which might be technically ineligible for implantation or SRS, can be safely and effectively treated. FSRT can be delivered with standard fractionation regimens or with hypofractionated schedules. The latter possibility is not only more beneficial to patients with respect to quality of life and convenience, but it may also represent a decrease in cost associated with retreatment.

Radiosurgery requires a single day of outpatient therapy, limiting treatment and hospitalization times. The main advantage of SRS is the capability of relevant dose delivery to the tumor volume while sparing surrounding normal tissues. This non-invasive approach enables the local application of radiation without surgical intervention. As a consequence, many of the risks involved with brachytherapy (such as infection, haemorrhage, exposure of the personnel to radiation) do not apply to radiosurgery. However, the potential advantage of radiation delivery over multiple cell-cycle times (as achieved in brachytherapy) is not provided. The argument for the use of radiosurgery is the relevant radiobiological effect of single-session radiation cell kill or cell division capability arrest, regardless of mitotic phase. Moreover, when the treatment volume is small and contains little functioning brain tissue, the need for fractionation may not apply. However, its high-dose focal radiation delivery may encounter in a high risk for side effects, when the treatment volume becomes larger or the tumor is at or close to eloquent structures (e.g., optic pathway, basal ganglia, speech or motor area). Due to the above-mentioned features, the application clinical setting is limited to patients with small, round shaped lesions and with good prognostic factors. However, also deep-sited lesions (usually considered not implantable) can be managed.

Brachytherapy also allows delivery of a large dose to the tumor volume while sparing surrounding normal tissue. However, the corresponding invasive procedures involve some surgical risks and require the patient's hospitalization. Considering that the radiation dose is usually delivered during 4 to 6 days, the radiobiological advantages include reoxygenation and the phase-specific cell sensitivity. Unfortunately, all BT implants generally produce inhomogenous dose distribution. Hence, the implantation of large tumors (even though feasible) should be avoided. Albeit the results obtained with this modality are encouraging, the technical complexity in performing brachytherapy implants limits its use in current clinical practice. Moreover, it could be offered to selected patients who are young and have good functional status and no-deep lesions.

Given that FSRT patients had comparable survival to SRS/BT patients, FSRT may be a better option for patients with larger tumors or tumors in eloquent structures.

The inherent variation of tumor and patient characteristics, as well as therapeutic interventions for recurrent HGG patients make comparison of patient groups from different studies unreliable. The series are mainly retrospective and feature several selection bias:

1. BT candidates had lobar tumors without involvement of midline structures, no ventricular disease, small tumor diameter and high KPS. Moreover, the surgical procedure allowed for maximal safe re-resection;
2. SRS series usually included patients with potentially adverse prognostic factors not amenable with BT;
3. Patients with larger recurrent tumors or tumor in eloquent structures were selected to receive FSRT compared with those treated with SRS/BT. As a consequence, potential prognostic variables predicting longer survival were preferentially distributed in favour of SRS/BT;
4. Many patients received additional and different therapies at the time of failure, making it difficult to properly interpret the results. In this regard, the end point of survival is a relatively poor measure of treatment efficacy, whereas time to failure after treatment is potentially less subject to the effects of selection bias. Unfortunately, time to failure data was generally not available for most series.

## 4. Conclusions

The standard of care for patients with recurrent HGG has not yet been clearly defined. Reoperation can only be performed in selected patients of younger age and with good performance status. Indeed, the infiltrative nature of the disease does not always allow a total resection without compromising neurologic functions.

The chemotherapy administration (especially TMZ), is probably the most frequent salvage treatment employed for recurrent HGG.

Considering the risk of acute and late side effects, re-irradiation of the same tumor site with conventional technique (EBRT) is considered to be troublesome.

Hypofractionated stereotactic radiotherapy as a salvage therapy is a non-invasive approach to deliver a precisely localized high radiation dose into a small volume. This technique is characterized by a steep dose fall-off to the periphery and may be a valuable re-treatment option after high-dose percutaneous radiotherapy. Further therapy options include BT and SRS but they are limited to smaller tumor volumes. In addition, BT and SRS show a higher risk of toxicities (e.g., radionecrosis).

Re-irradiation using these high precision techniques allows the survival prolongation and delays disease progression or recurrence. However, it is not a curative treatment and it is limited to selected subgroups of patients. Therefore, a further therapeutic improvement is needed. The radiochemotherapy combination as well as alternative treatment modalities are worthy of investigation.

## Figures and Tables

**Table 1.** Re-irradiation with external beam radiotherapy with or without chemotherapy.

**Author [ref.]**	**≠ Pts**	**Med. age (years)**	**Med. KPS**	**Surgery before re-irr.**	**Med. total dose (Gy)**	**Med. time to re-irr. (mos.)**	**Med. re-irr. total dose (Gy)**	**CHT**	**Med. survival (mos.)**	**Neurol. side effects**

Bauman *et al.* [11]	10	20	80	1/10	54– 72	15.5	18–74	5/10 RT+CHT (various)	OS 8.3 PFS 3.3	RN 3 Deterioration 2 Reop 3
Kim *et al.* [12]	14 (7 GBM)	21–52	60	7/14	59.4	38	36	RT alone	OS 7	RN 1
Hayat *et al.* [13]	11	41	80	NR	45	31	30/2.5	RT+CHT (CCNU)	OS 13	Cort prolonged
Arcicasa *et al.* [14]	24	44	70	4 total 8 subtot	60	14	34.5	RT+CHT (CCNU)	OS 13.7	No
Nieder *et al.* [15]	32 (21 GBM)	44	70	14	58.6	20	45.5 bid	RT alone	OS 8.5 PFS 5	Reop 5 RN 2 Late neurotox. 15%
Verninga *et al.* [16]	22 (17 GBM)	34	WHO ≤ 2	21/42 (+other)	60	32.8	46	RT alone	OS GBM 6.1 HGG 8.2	RN 1 Cognitive decline 1 Cort 8/10 increased, 18/32 started
Henke *et al.* [17]	31 (29 GBM)	50	90	16/31 12 total	59	NR	20	15/31 RT+CHT (various)	OS 10.2	RN 2 Reop 6

GBM: glioblastoma; Med.: median; NR: not reported; Gy: Gray; bid: bis in die; OS: overall survival; PFS: progression-free survival; HGG: high-grade glioma; re-irr.: re-irradiation; CHT: chemotherapy; mos.: months; CCNU: lomustine; KPS: Karnofsky performance status; Cort: corticosteroids; Neurol.: neurologic; RN: radionecrosis; Reop: reoperation.

**Table 2.** Re-irradiation with fractionated stereotactic radiation therapy with or without chemotherapy.

**Author [ref. ]**	**≠ Pts.**	**Med. age (years)**	**Med. KPS**	**Surgery before re-irr.**	**Med. total dose (Gy)**	**Med. time to re-irr. (mos.)**	**Med. re-irr. total dose (Gy)**	**Med. vol. (cc)**	**CHT**	**Med. surv. (mos.)**	**Neurol. side effects**

Laing *et al.* [18]	19 (12 GBM)	34	70	6 STR	55	20	40	25	--	OS 9.8	5 neurol. deterioration
Glass *et al.* [19]	20 (13 GBM)	44	90	NR	NR	8	42	14.3	Cis-DDP	OS 13.7	3 RN 1 somnolence 1 confusion 1 seizure
Shepherd *et al.* [20]	29	37	80	6/29	55	29	20–50	24	--	OS 10.7	4 RN 2 Reop 1 steroid increase
Lederman *et al.* [21]	14 (GBM)	56	70	NR	60	7.8	24	32.7	TAX	OS 7	7 RN 11 Reop
Hudes *et al.* [22]	20 (19 GBM)	52	80	NR	60	3.1	24–35	12.6	--	OS 10.5	3 steroid increase
Cho *et al.* [23]	20 (15 GBM)	53	60	NR	59.4	19	37.5	25	--	OS 12	1 RN 3 Reop 41 steroid start
Selch *et al.* [24]	21 (14 GBM)	54	80	4 STR	60	11	25	12	--	OS 6.7 PFS 4	No
Voynov *et al.* [25]	10 (4 GBM)	48	80	5/10 STR	59.7	19	30	34.7	5/10 various	OS 10.1	2 Reop 6 RN
Combs *et al.* [26]	40	42	≥80: 38	9 STR	59.4	34.5	36	56.2 (PTV)	--	OS 16 PFS 8	No > G2
Combs *et al.* [27]	50 (GBM)	55	≥80: 46	NR	57	10	36	49 (PTV)		OS 8 PFS 5	No > G2
Vordemark *et al.* [28]	19 (14 GBM)	50	90	12/19	45– 61	19	30	15	--	OS 9.3 GBM 7.3 HGG 15.4 PFS 4.9	1 reop
Grosu *et al.* [29]	44 (33 GBM)	50	80	NR	60	16	30	15	29/44 TMZ	OS 8	6 steroid increase
Wurm *et al.* [30]	25 (20 GBM)	45	80	NR	54.4 bid/60	12.8	25–30	16.5	Topo	OS 14.5 PFS 10.5 (PFS6 42%)	3 G2 RTOG
Kohshi *et al.* [31]	25 (11 GBM)	46	70	NR	60	11	28.1– 68.2	8.7	--	OS GBM 11 HGG 19	7 Reop (partial RN)
Ernst-Stecken *et al.* [32]	15 (11 GBM)	49	80	NR	57.75	10	35	5.7	--	1 year OS 43% PFS 53% (PFS6 75%)	3 steroid increase
Schwer *et al.* [33]	15 (11 GBM)	47	70	4 GTR 3 STR	60	11.9	18–36	41.3	Gef	OS 10 PFS 7 (PFS6 63%)	3 seizure 2 deterioration
Combs *et al.* [34]	25 (GBM)	39	≥70 92%	5 GTR 13 STR	60	36	36	50	TMZ	OS 8 PFS 5 (PFS6 48%)	No
Fokas *et al.* [35]	53 (GBM)	53	70	23/53	54	NR	30	35	--	OS 9	No
Patel *et al.* [36]	10 (GBM)	44	90	2 GTR 4 STR	50– 60	14.9	36	51.1	--	OS 7.4	1 RN 1 Reop

Gutin *et al.* [37]	25 (20 GBM)	56	80	NR	59.4	15	30	34	Beva	OS 12.5 PFS 7.5 (PFS6 64%)	3 Reop 1 hemorrhage 1 wound deiscence
Villaceincio *et al.* [38]	26 (GBM)	56	80	15 GTR 9 STR	59.4	13	20	7	--	OS 7	NR
Fogh *et al.* [39]	147 (105 GBM)	NR	NR	24 GTR 60 STR	60	9	35	22	48/147 various	OS GBM 11 HGG 10	15 steroid increase
Torcuator *et al.* [40]	16	55.7	90	No	NR	NR	36	NR	12/16 Beva+TMZ or other	OS 7.2 PFS 2.6	No
Minniti *et al.* [41]	36 (GBM)	56	70	NR	60	14	37.5	13.1	TMZ	OS 9.7 PFS 3 (PFS6 42%)	3 RN
Hauff *et al.* [42]	59 (GBM)	55.7	90	11/59	Dose NR	NR	30 + HT	46.5	--	OS 13.4	No

Pts.: patients; GBM: glioblastoma; NR: not reported; tot: total; subtot: subtotal; vol.: volume; mos.: months; GTR: gross total resection; STR: subtotal resection; RN: radionecrosis; re-irr: reirradiation; Gy: Gray; OS: overall survival; PFS: progression free survival; PFS6: 6-month progression-free survival; CHT: chemotherapy; KPS: Karnofsky performance status; RN: radionecrosis; Reop: reoperation; TMZ: temozolamide; cis-DDP: cisplatin; Beva: bevacizumab; Topo: topotecan; Gef: gefitinib; TAX: paclitaxel; HT: thermotherapy; G: grade; RTOG: Radiation Therapy Oncology Group; PTV: planning target volume.

**Table 3.** Series of high-grade gliomas treated by stereotactic radiosurgery.

**Author [ref. ]**	**≠ Pts.**	**Med. age (years)**	**Med. KPS**	**Med. TD before re-irr. (Gy)**	**Med. re-irr. TD (Gy)**	**Med. time to re-irr. (mos.)**	**Med. Vol. (cc)**	**Med. survival from re-irr. (mos.)**	**Neurol. side effects**

Alexander *et al.* [43]	25 (16 GBM)	45	80	59.4	13 to 70% isodose (Linac)	14	10	OS 9	RN 12%
Chamberlain *et al.* [44]	13 (5 GBM)	Mean 42 (GBM) Mean 34 (HGG)	Mean 60 (GBM) Mean 80 (HGG)	Mean 62 (GBM Mean 58 (HGG)	Mean 12 (GBM) Mean 14 (HGG) (Linac)	Mean 10.4 (GBM) Mean 36.3 (HGG)	Mean 34.3 mL (GBM) Mean 16 mL (HGG)	OS 8 * PFS 4 *	NS
Shrieve *et al.* [45]	86 (GBM)	46	80	NR	13 to med. 80% isodose (Linac)	10.3	10.1	OS 10.2	Seizures 3.5%hosp. 2.5% exitus 1% cr. nerve deficit 1% Reop 22%
Larson *et al.* [46]	63 (46 GBM)	53 (GBM) 43 (HGG)	90	NR	Med. min. 16 to med. 50% isodose (GK)	>16 weeks	6.2 (GBM) 7.5 (HGG)	OS 57 weeks (GBM) not reached (HGG; 1y OS 68%)	NR
Kondziolka *et al.* [47]	42 (19 GBM)	Mean 51 (GBM) Mean 45 (HGG)§	Mean 90§	Mean 60	Mean 15.5 (GBM) Mean 15.2 (HGG) to 50% isodose (GK)	18.9 (GBM) 19.8 (HGG)	Mean 6.5 mL (GBM) Mean 6 mL (HGG)§	OS 30 (GBM) 31 (HGG)	Reop 19% RN 1.6%(GBM) Reop 23% RN 4.7% (HGG)§

Cho *et al.* [23]	46 (27 GBM)	48	70	60	17 to med. 50% isodose (Linac)	10	30 mL	OS 11	Initiation or steroids increase 41% Reop 14% RN 4.3%
Park *et al.* [48]	23 (GBM)	53	80	NR	15 to 60% isodose (Linac/GK)	NR	9.9	OS 10.3 PFS 4.7	NR
Larson *et al.* [49]	26 (14 WHO 4)	53 (WHO 4) 44 (HGG)	90	NR	Med min. 15 (WHO 4) 16.5 (HGG)(GK) #	12 (WHO 4) 43 (HGG)	8 (WHO 4) 2.7 (HGG)	OS 38 weeks PFS 15 weeks (WHO 4) OS 68 weeks PFS 29 weeks (HGG)	NR
Combs *et al.* [50]	32 (GBM)	56	≥70	54	15 to 80% isodose (Linac)	10	10 mL	OS 10 PFS 5 (PFS6 33%)	No > G2 (acute)
Hsieh *et al.* [51]	26 (GBM)	58	Mean 70	60	12 to 50% isodose (GK)	NR	Mean 21.6	OS 10	RN 31.3% §
Mahajan *et al.* [52]	41 (GBM)	54	90	60	NR (Linac)	10	4.7	OS 11	Reop 22%
Kong *et al.* [53]	114 (65 GBM)	49	80	60	16 to 50% (GK) or 80% (linac) isodose (Linac/GK)	NR	10.6 mL	OS 13 PFS 4.6 (GBM) OS 26 PFS 8.6 (HGG)	Reop 3.5% RN 24%
Biswas *et al.* [54]	18 (GBM)	57.8 §	≥70	60	15 to the isocenter (Linac)	12.1	8.4 mL	OS 5.3 PFS 3.4	No > G2 (acute)
Patel *et al.* [36]	26 (GBM)	53	80	Range 50-60	18 to 90% isodose (Linac) ¶	12	10.4	OS 8.4	NS
Maranzano *et al.* [55]	13 (GBM)	55	90	60	17 to the isocenter (Linac)	9	5.3	OS 11	RN 23%

Med.: median; GBM: glioblastoma; HGG: high-grade glioma; surg.: surgery; TD: total dose; re-irr.: re-irradiation; min.: minimum; mos.: months; GK: gamma-knife; Linac: linear accelerator; NR: not reported; NS: not specified; OS: overall survival; PFS: progression-free survival; PFS6: 6-month progression-free survival; Reop: reoperation; hosp: hospitalization; cr.: cranial; RN: radionecrosis; Gy: Gray; CHT: chemotherapy; KPS: Karnofsky performance status; Neurol.: neurological;

°: some patients included in different publications;

*: this series also includes some LGG;

§: data refer both to newly and recurrent HGG;

#: delivered before marimastat (10 mg b.i.d.);

¶: delivered before not specified chemotherapy.

**Table 4.** Series of high-grade gliomas treated by brachytherapy.

**Author [ref. ]**	**≠Pts**	**Med. age (years)**	**Med. KPS**	**Surg. before re-irr. (%)**	**Med. TD before re-irr.(Gy)**	**Med re-irr. TD (Gy)**	**Med. interval to re-irr. (mos.)**	**Med. volume (cc)**	**Med. survival from re-irr. (mos.)**	**Side effects**

Lucas *et al.* [56]	20 (7 GBM)	Mean 43 (GBM) Mean 32 (HGG)	NR	NR	Mean 58.2	Mean 50 Temp HDR 192-Ir sources	NR	NR	OS 10 (GBM) OS 18 (HGG)	Seizures 6% wound inf 6% CSF leak 3% Reop 9% RN 3%
Scharfen *et al.* [57]	111 (65 GBM)	Mean 46§	90 §	NR	60 §	64.4 Temp LDR 125-I sources	NR	NR	OS 49 weeks (GBM) OS 52 weeks	G3 6% G4 1% G5 < 1% (Acute) § Reop 38% (GBM) 47% (HGG) RN 5% §

Malkin *et al.* [58]	36	NR	Mean 75	NR	NR	60 Temp LDR 125-I sources	NR	Mean 41	OS 10	Misplacement 9% Reop 43% RN 9%
Sneed *et al.* [59]	42 (26 GBM)	43	90	NR	NR	Range 50-60 Temp LDR 125-I sources + HT	NR	17	OS 47 weeks (GBM) OS not achieved 1y OS 81% (HGG)	Reop 43% RN 5%
Bernstein *et al.* [60]	44 (32 GBM)	46	≥60	NR	Range 50–60	Mean 70.17 Temp LDR 125-I sources	12.5	50.3	OS 46 weeks *	IPI 6.5%catheter migration 2.1% (Acute) steroid-dep. 90% Reop 26.1% RN 4.3%
Kitchen *et al.* [61]	23	Mean 45	Mean 69	NR	NR	50 (DP) Temp LDR 125-I	51 weeks	Mean 28	OS 25 weeks	NS
Shrieve *et al.* [45]	32 (GBM)	45	80	NR	NR	50 Temp LDR 125-I sources	7.3	29	OS 11.5	Scalp inf 6% (Acute) visual deficit 6% Reop 44% RN 6%
Chamberl ain *et al.* [62]	15	47	NR	NR	NR	50 ¶ Temp LDR 125-I sources	NR	25	31%PR 44%SD 25% P	Steroid-dep. 62% dementia 6% Reop 56% RN 56%
Mayr *et al.* [63]	41 (28 GBM)	52 §	80 §	NR	NR	Mean 59 § Temp LDR 125-I sources	NR	Mean 28 §	41.2 weeks (GBM) 32.6 weeks (HGG)	RN 16% other compl. 13%
Simon *et al.* [64]	42 (GBM)	49	80	B 100	Range 46–60	50 Temp LDR 192-Ir sources	NR	23	OS 50 weeks	Skin necrosis 4.7%men. 9.5% Reop 24% RN 7%

Tselis *et al.* [65]	84 (GBM)	57	80	NR	Up to 60	40 Temp HDR 192-Ir sources	NR	51	OS 37 weeks	Intracerebral bleeding 2.3%men. 1.1% RN 2.3% (Acute)
Fabrini *et al.* [66]	21	60	80	GTR/ STR 100	60 (all pts)	18 Temp HDR 192-Ir sources	NR	NR	OS 5.5	G1 headache 100% G2 CFS leak 4.7% G3 CFS leak 4.7% G5 4.7% (Acute) asymptomatic RN 9.5%
Larson *et al.* [67]	33 (13 GBM)	55 (GBM) 40 (HGG)	NR	Max. safe res. 100	NR	Range 40–50 Perm LDR 198-Au seeds	NR	NR	OS 9 (GBM) OS 17 (HGG)	No
Halligan *et al.* [68]	21	NR	≥50	GTR 86 STR 14	Range 54–64.8	210 (DP) Perm LDR 125-I seeds	NR	NR	OS 65 weeks PFS 29 weeks	No
Gaspar *et al.* [69]	60 (37 GBM)	47	≥60	Max. safe res. 92 B 8	Range 50–66	103.68 (DP) Perm LDR 125-I seeds	NR	17	OS 10.8 (GBM) OS 24.4 (HGG)	Reop 40% RN 5%
Patel *et al.* [70]	40 (GBM)	50	70	GTR 55 STR 45	60 (all pts)	Range 120-160 Perm LDR 125-I seeds	NR	47.3	OS 47 weeks PFS 25 weeks	Healing compl. 5%
Larson *et al.* [71]	38 (GBM)	47	90	STR 60 (resid. ≥0.5 cm) STR 40 (resid. <0.5 cm)	60 (all but 2 pts)	300 Perm LDR 125-I seeds	39 weeks	21 Pre-implant	OS 52 weeks PFS 16 weeks	Reop 10.5% RN 2.6%

Darakchiev *et al.* [72]	34 (GBM)	53	80	GTR 85 STR 15	NS	120 (DP) @ Perm LDR 125-I seeds	NR	34 before surgery	OS 69 weeks PFS 47 weeks (PFS6 67%)	Healing compl. 11.7% Reop 29.4% RN 23.5%
Tatter *et al.* [73]	21 (15 GBM)	Mean 48.4	80	Max. safe res. 100	NS	Range 40–60 GliaSite-Iotrex	NR	NR	OS 8 (GBM) OS 17.9 (HGG)	Pseudo-meningocele 4.7%wound inf 4.7%chemical men.4.7%
Chan *et al.* [74]	24 (GBM)	48	80	Max. safe res. 100	Mean 59.8	Mean 53.1 GliaSite-Iotrex	NR	NR	OS 9.1	G1-2 headache 42%nausea-vomiting 4%wound inf 6%(Acute) neurological deficit 4%RN 8%
Gabayan *et al.* [75]	95 (80 GBM)	51	80	Max. safe res. 100	60	60 GliaSite-Iotrex	40.6 weeks	NR	OS 35.9 weeks (GBM) OS 43.6 weeks (HGG)	G1 1.1% G2 8.4% G3 2.1% (RN)

Med.: median; GBM: glioblastoma; HGG: high-grade glioma; surg.: surgery; TD: total dose; re-irr.: re-irradiation; mos.: months; NR: not reported; NS not specified; OS: overall survival; PFS: progression-free survival; PFS6: 6-month progression-free survival; reop: reoperation; RN: radionecrosis; IPI: intracranial pressure increase; inf: infection; compl.: complication; men.: meningitis; dep.: dependent; Gy: Gray; CHT: chemotherapy; KPS: Karnofsky performance status; DP: dose prescription; max.: maximal; res.: resection; GTR: gross total resection; STR: sub-total resection; B: biopsy; BCNU: carmustine; Perm: permanent; Temp: temporary; LDR: low-dose rate; HDR: high-dose rate; HT: hyperthermia; CSF: cerebrospinal fluid; pts: patients; I: iodium; Ir: iridium; C.E.: contrast enhancement.

*: data also include 2 LGGs;

§: data refer both to newly diagnosed and recurrent HGG;

¶: delivered in combination with cisplatin (20 mg/m^2^ × 5 gg);

@: delivered in combination with BCNU wafers (Med. 61.6 mg).
